# Transcriptomic analysis of pancreatic adenocarcinoma specimens obtained from Black and White patients

**DOI:** 10.1371/journal.pone.0281182

**Published:** 2023-02-22

**Authors:** Thomas G. Biel, Svetlana Petrovskaya, Francesca Mascia, Tongzhong Ju, Lola Fashoyin-Aje, Kelly M. Herremans, Andrea N. Riner, Patrick W. Underwood, Michael H. Gerber, Martha Donoghue, Jose G. Trevino, V. Ashutosh Rao

**Affiliations:** 1 Office of Biotechnology Products, Center for Drug Evaluation and Research, Food and Drug Administration, Silver Spring, Maryland, United States of America; 2 Office of Oncologic Diseases, Office of New Drugs, Center for Drug Evaluation and Research, Food and Drug Administration, Silver Spring, Maryland, United States of America; 3 Department of Surgery, College of Medicine, University of Florida, Gainesville, Florida, United States of America; Kyushu University Hospital: Kyushu Daigaku Byoin, JAPAN

## Abstract

In pancreatic cancer clinical trials, Black patients are under-represented while having higher morbidity and mortality rates as compared to other racial groups. Multiple factors, including socioeconomic and lifestyle factors may contribute to this disparity, but genomic contributions remain unclear. In an exploratory project to identify genes that may contribute to differences in survival between Black (n = 8) and White (n = 20) patients with pancreatic cancer, transcriptomic sequencing of over 24,900 genes was performed in human pancreatic tumor and non-tumor tissue obtained from Black and White patients. Over 4,400 genes were differentially expressed in tumor and non-tumor tissue, irrespective of race. To validate these results, the expression of four genes (AGR2, POSTN, TFF1, and CP) reported to be up-regulated in pancreatic tumor tissue as compared to non-tumor tissue were confirmed using quantitative PCR. Transcriptomic analysis that compared pancreatic tumor tissue from Black and White patients revealed differential expression in 1,200 genes, while a comparison of the non-tumor and tumor gene expression differences within each race revealed over 1,500 tumor-specific differentially expressed genes in pancreatic tumor and non-tumor tissue from Black patients. We identified *TSPAN8* as a potential tumor-specific gene significantly overexpressed in pancreatic tumor tissue in Black patients as compared to White patients. Using Ingenuity Pathway Analysis software to compare the race-associated gene expression profiles, over 40 canonical pathways were identified to be potentially impacted by the gene expression differences between the races. Heightened expression of TSPAN8 was associated with poor overall survival, suggesting TSPAN8 as one potential genetic factor contributing to the differential outcomes in Black patients with pancreatic cancer, supporting the potential utility of larger genomic studies to further explore the role of TSPAN8 in pancreatic cancer.

## Introduction

Pancreatic cancer has the lowest 5-year survival rate of all the major organ cancers and an increasing incidence in the population [1, 2]. By 2040, pancreatic cancer is projected to surpass colorectal carcinoma to become the second most common cause of cancer-related death [2]. Racial disparities in pancreatic cancer morbidity and mortality between Black and White patients in the United States have been previously described based on data from the National Program of Cancer Registries and the Surveillance, Epidemiology, and End Results (SEER) program [1, 3–5]. Among all age groups, from 2001 through 2015, Black patients in the United States experienced a higher pancreatic cancer incidence (24.1 vs 19.4 per 1,000,000) and higher mortality (23.3 vs 19.4 per 1,000,000) compared to White patients [4]. Socioeconomic, lifestyle factors, and comorbidities alone may not fully account for these disparities [5–8], suggesting that underlying differences in tumor biology could play a role in differences in outcome. The lack of adequate representation of Black patients in clinical trials and in tumor genetic databases may also contribute to the observed disparities. For example, in the United States, the majority of the participants in pancreatic cancer clinical studies between 2005 to 2020 were White (82%) while only a small proportion were Black (8.2%) [3].

Biospecimens from Black patients required to generate an inclusive and representative pancreatic cancer genetic database are limited [9]. For instance, as of March 2022, the cancer genome atlas, a biobanking program, reports only 2.2% of the 2668 pancreatic cancer cases were collected from Black patients [10]. Most information on susceptibility to pancreatic cancer is based on data from White patients, and the contribution of gene expression variation for other racial groups is unclear [5]. While the causes of racial disparity in outcomes of patients with pancreatic cancer are likely to be multi-factorial, identification of potential genetic factors that may contribute to differential outcomes in Black and White patients may guide future work and approaches to development of targeted treatments that can improve outcomes in Black patients with pancreatic cancer [7, 11].

Using pancreatic tumor and non-tumor tissue from Black and White patients, we performed an exploratory investigation of transcriptomic factors that may contribute to disparities in pancreatic cancer outcomes across these two populations. In this report, the gene expression profiles of pancreatic non-tumor and tumor tissues from Black and White patients were investigated to demonstrate the importance of diversifying and expanding genetic databases to support pancreatic cancer research and precision medicine efforts. Using the dataset generated in our study, a tissue-specific comparative analysis was performed which revealed specific genes were significantly upregulated in pancreatic tumor tissue specimens obtained from Black patients, as compared to non-tumor pancreatic tissue in Black patients and, compared to either tissue type in White patients; this may suggest these genes as potential \pancreatic cancer biomarkers that may be enriched in Black patients. Using Ingenuity Pathway Analysis (IPA) software, the tumor-specific gene expression profiles were compared to identify potential canonical pathways differences in pancreatic cancer tissue specimens from Black and White patients.

## Materials and methods

### Human pancreatic tissue

This study involved the use of existing de-identified tumor specimens and all procedures related to the collection and testing of human tissue adhered to IRB-approved protocols at the University of Florida and the United States Food and Drug Administration. Study methodologies were approved by IRB committees and conformed to the standards set by the Declaration of Helsinki. Written informed consent was obtained from adult patients participating in the study. Tumor and non-tumor tissues were collected, and flash frozen at the University of Florida during pancreatic resection surgery from patients that self-identified as “black” or “white”. All but two of the patients were treatment-naïve prior to tumor resection.

### Nucleic acid extraction from tissue

Approximately 25 to 50 mg of frozen tissue was placed in RNA later-ICE (Thermofisher) and thawed for 12 hours at -20°C. A RNeasy mini kit (Qiagen) was utilized to extract the nucleic acid as described by the manufacturer’s protocol.

### Transcriptomic analysis

RNA library preparation was performed using the Illumina TruSeq Stranded Total RNA Kit according to the manufacturer’s protocol. The library preparation included ribosomal RNA removal, RNA fragmentation using alternate protocol, cDNA synthesis, adaptor ligation, and cDNA amplification. Input RNA and intermediate and final DNA products were checked on the TapeStation 4200 (Agilent) and Qubit (Invitrogen). 2x100 bp paired-end sequencing was run on the Illumina NextSeq 500 using High Output Kit v2 (300 cycles).

Resulting sequencing reads were quality-checked and analyzed using Homo sapiens/hg19 (RefSeq) reference genome and TopHat Alignment software v1.0.1 on the BaseSpace Onsite Hub (BSO, Illumina). Gene pathway analysis was performed using Ingenuity Pathway Analysis (Qiagen).

### Quantitative real time polymerase chain reaction (Q-RT-PCR)

RNA sequencing data were verified on the same samples with Q-RT-PCR using Power SYBR Green Mix (Thermo Fisher Scientific) and Qiagen QuantiTect Real Time optimized primer assays. RNA was reverse transcribed using the High-Capacity cDNA reverse transcription kit (Thermo Fisher Scientific). Q-RT-PCR was performed on a QuantStudio 6 Flex (Thermo Fisher Scientific) using the conditions recommended by the manufacturer. Data are plotted as fold change over basal level with relative expression obtained with Delta Ct method normalized with *PMM1* as housekeeping gene.

### Analysis of differential gene expression

Significant (*p*<0.05) gene expression differences identified from the pancreatic non-tumor and tumor tissue were analyzed using IPA CORE analysis [12]. These results were then compared to identify similarities, differences, and trends in canonical pathways using IPA Comparison analysis. Assuming the data’s normally distributed, a z score cutoff of 1.7 was applied to identify canonical pathways that were significantly different between the pancreatic tumor tissue between Black and White patients.

### Reagents, overall survival and statistics

Unless described otherwise, all reagents were purchased from Sigma Aldrich. Statistical analyses were performed using GraphPad Prism 6, except for the statistics used to compare overall patient survival in the Kaplan-Meier plots. When comparing two groups, a Student’s T test was performed with and without a 1% false discovery rate (FDR). When 1 factor was analyzed, a one-way ANOVA was performed with a Sidak’s multiple comparison test to identify statistical differences. When >1 factor was analyzed, a two-way ANOVA was performed with a Tukey’s multiple comparison post-hoc test to identify significant differences. A nominal *p* value of < 0.05 indicated statistical differences. The degree of freedom, *p* values, and test methods are provided in S1 Table. Kaplan-Meier plots and a log-rank test to analyze survival differences were generated using a Kaplan-Meier plotter software as previously described [13]. The Kaplan-Meier software identified the cut-off values for each gene using predefined quantiles, trichotomizing the data, and using the best available cut-off value with quality control as previously described [13, 14].

## Results

### Gene expression differences in human pancreatic tumor and non-tumor tissues

**Fig 1:**
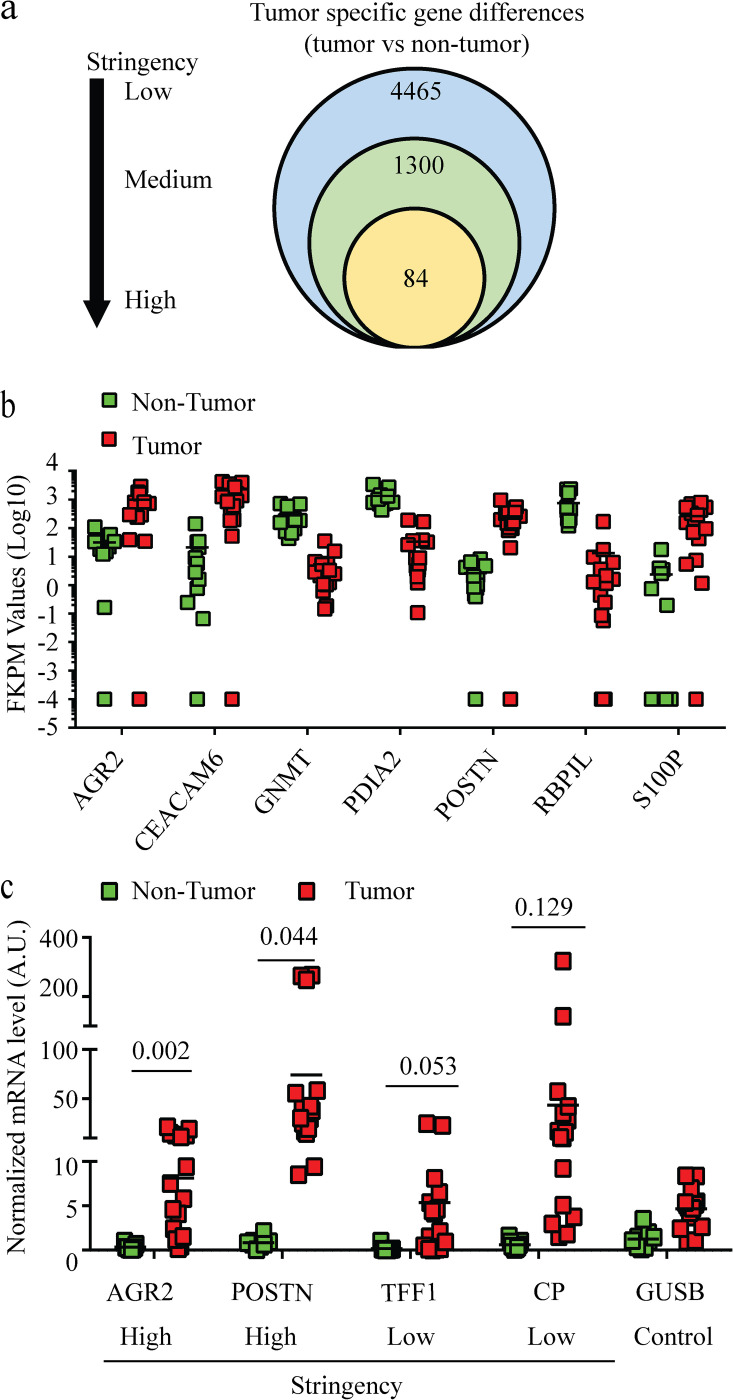
Characterization of human pancreatic tumor tissue transcriptome as compared to the non-tumor tissue transcriptome. (a) Venn diagram of the number of significant gene differences that meet the low, medium, and high stringency criteria form the dataset. (b) High stringency identified genes that were statistically different (nominal *p*<0.05) between tumor and non-tumor pancreatic tissue using a two-tailed ANOVA with tissue type and gene as factors. (c) mRNA levels of *AGR2*, *POSTN*, *TFF1*, *CP and PMM1* using RT-PCR. *PMM1* was used as a control. The *p* values from each Student T test are provided for each gene and the mean values are presented as bars.

To identify specific gene expression differences in pancreatic tumor compared to non-tumor tissue, the transcriptomic profiles of 16 tumor and 12 non-tumor tissues from different patients were characterized and compared using next generation RNA sequencing (NGS) (Table 1 and S2 Table). The expression levels of over 24,900 genes were established for each of the 28 pancreatic tissue specimens (S1 File). Among the studied genes, there were over 4,000 genes with statistically significant differences in expression between pancreatic tumor and non-tumor tissue, using a Student’s T test with a 0.1% FDR (low stringency criterion) (Fig 1A). To increase the stringency of the genetic analysis, two cutoff intervals were applied, i.e., 2 < Log_2_ < -2 (medium stringency criterion) and 5 < Log_2_ < -5 (high stringency criterion) (S2 Table). This reduced the number of differentially expressed genes to 1,300 and 84, respectively (Fig 1A and S3 Table). Using a two-way ANOVA with tissue type and gene as factors, the individual tissue gene expression levels of the 84 genes that met the high stringency criterion were compared to identify a statistical interaction effect between the gene and tissue type (S1 Fig); in this analysis, only 7 of the 84 genes had expression levels that were identified to be significantly different between the pancreatic tumor and non-tumor tissue (*AGR2*, *CEAMAN6*, *GNMT*, *PDIA2*, *POSTN*, *RBPJL*, and *S100P)* (Fig 1B).

**Table 1:** Demographic information of the pancreatic tissue collected.

*Demographic Mean Age*				
Subjects	28		Years of Age [Ave. +/- S.D.]	58 +/- 15.45
*Tissue and Gender Cohorts*				
*Classification*	* *	*Race*	*Subjects*	*Years of Age [Ave*. *+/- S*.*D*.*]*
*Tumor*	*Total*	* *	*16*	*68 +/- 8*
		White	11	69 +/- 7
		Black	5	69 +/- 11
*Non-Tumor*	*Total*	* *	*12*	*46 +/- 13*
* *		White	9	48 +/- 11
		Black	3	39 +/- 18

Ave. = average, S.D. = Standard Deviation

To validate the NGS transcriptomic profiling, mRNA of the two genes identified to be significantly different at both the lowest and highest stringency levels were quantified using RT-PCR (Fig 1C). *AGR2*, *POSTN*, *TFF1*, and *CP* were selected because their transcribed proteins have been previously implicated as potential biomarkers for pancreatic cancer [15–18]. *AGR2* and *POSTN* expression, which met the high stringency criterion, were confirmed to be significantly elevated in tumor tissue compared to non-tumor tissue, while *TFF1* and *CP*, which met the low stringency criterion, had an increasing trend in expression in tumor tissue compared to non-tumor tissue, that did not meet statistical significance.

### Subcellular localization of pancreatic tumor specific gene products

**Fig 2:**
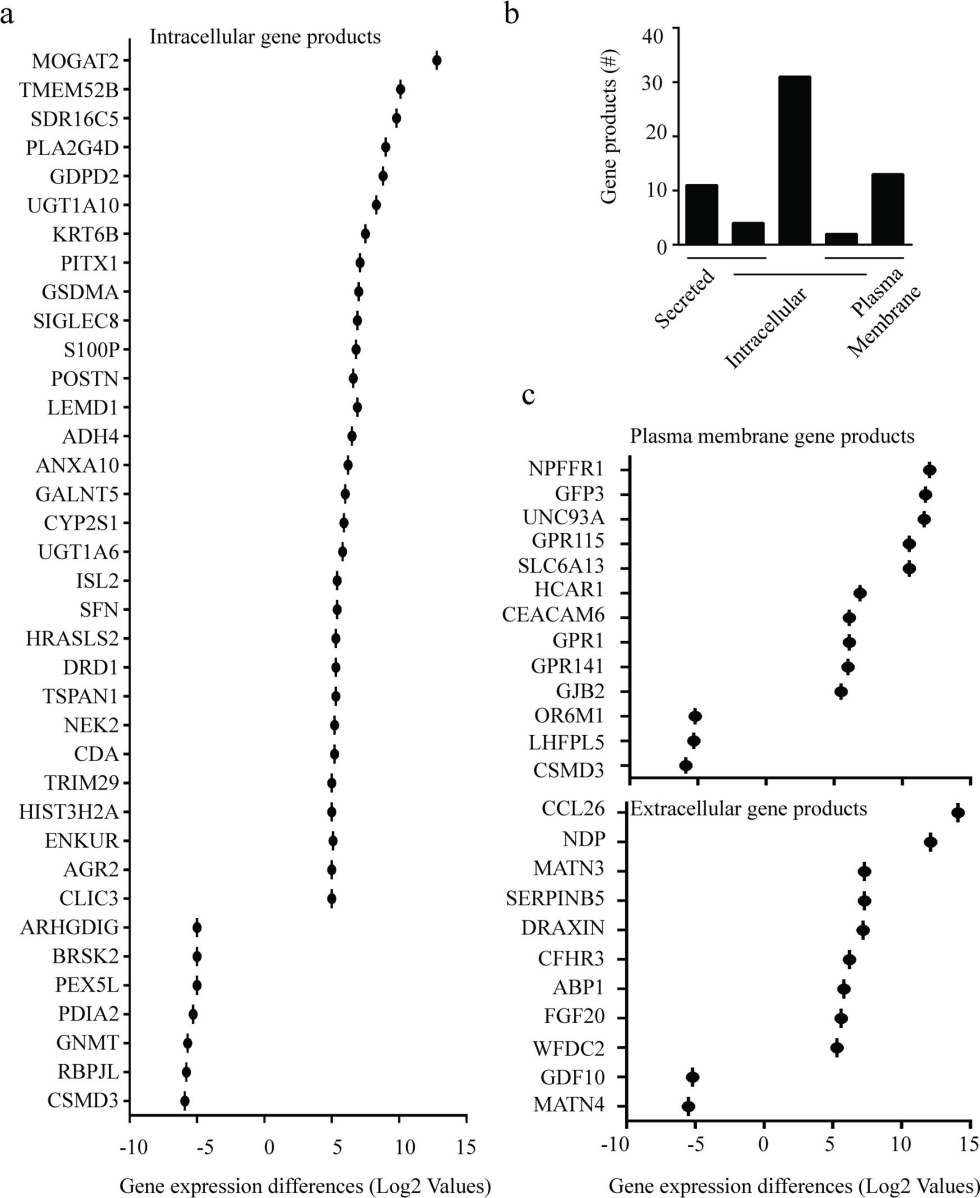
Genes within the high stringency criterion for differential expression between tumor and non-tumor pancreatic tissue and the subcellular localization of theoretical protein products. (a) Log fold differences of genes identified to translate intracellular proteins. (b) Quantity of gene products that were localized to the intercellular, extracellular (secreted), and/or within the plasma membrane. (c) Log fold differences of genes that translate proteins localized to the plasma membrane or extracellular space.

The subcellular distribution of protein biomarkers for cancer prognostics (tissue-based proteins) and diagnostics (blood- and serum-based proteins) is an emerging field that strives to deliver protein biomarkers that can be more practically translated to the clinic based on ease of tissue sampling [19]. Many promising protein-based diagnostics use proteins that are located extracellularly in the blood or serum [20], while tissue-based protein biomarkers have been primarily used to characterize the tumor tissue for prognostics and to inform therapeutic strategies [19]. To support the development of subcellular distribution protein-based diagnostics and prognostics, we analyzed our data set using Ingenuity Pathway Analysis (IPA). For the 84 identified genes that met the high stringency criterion from our dataset, the theoretically expressed proteins were localized to the plasma membrane and intercellular space (tissue-based proteins) or the extracellular space (plasma- and serum-based proteins) [12]. The IPA-identified protein locations were then confirmed using the predefined protein location from the Uniprot database [21]. Among the theoretically translated proteins, the location of 24 proteins failed this confirmation exercise (28%) due to limited information or discrepancies in the cellular localization between the databases as shown in S4 Table. Using the 60 theoretically expressed proteins that were confirmed by the Uniprot database, 37 were localized to the intracellular space (Fig 2A). The theoretically expressed proteins from *POSTN*, *AGR2*, *ANXA10*, and *CDA* were also identified to be extracellular, while the proteins from *ISL2* and *GDPD2* were also localized to the plasma membrane (Fig 2B and S5 Table). A total of 13 theoretically expressed proteins were localized to the plasma membrane, while 11 were secreted (Fig 2C).

### Gene expression differences between pancreatic non-tumor and tumor tissues from Black and White patients

**Fig 3:**
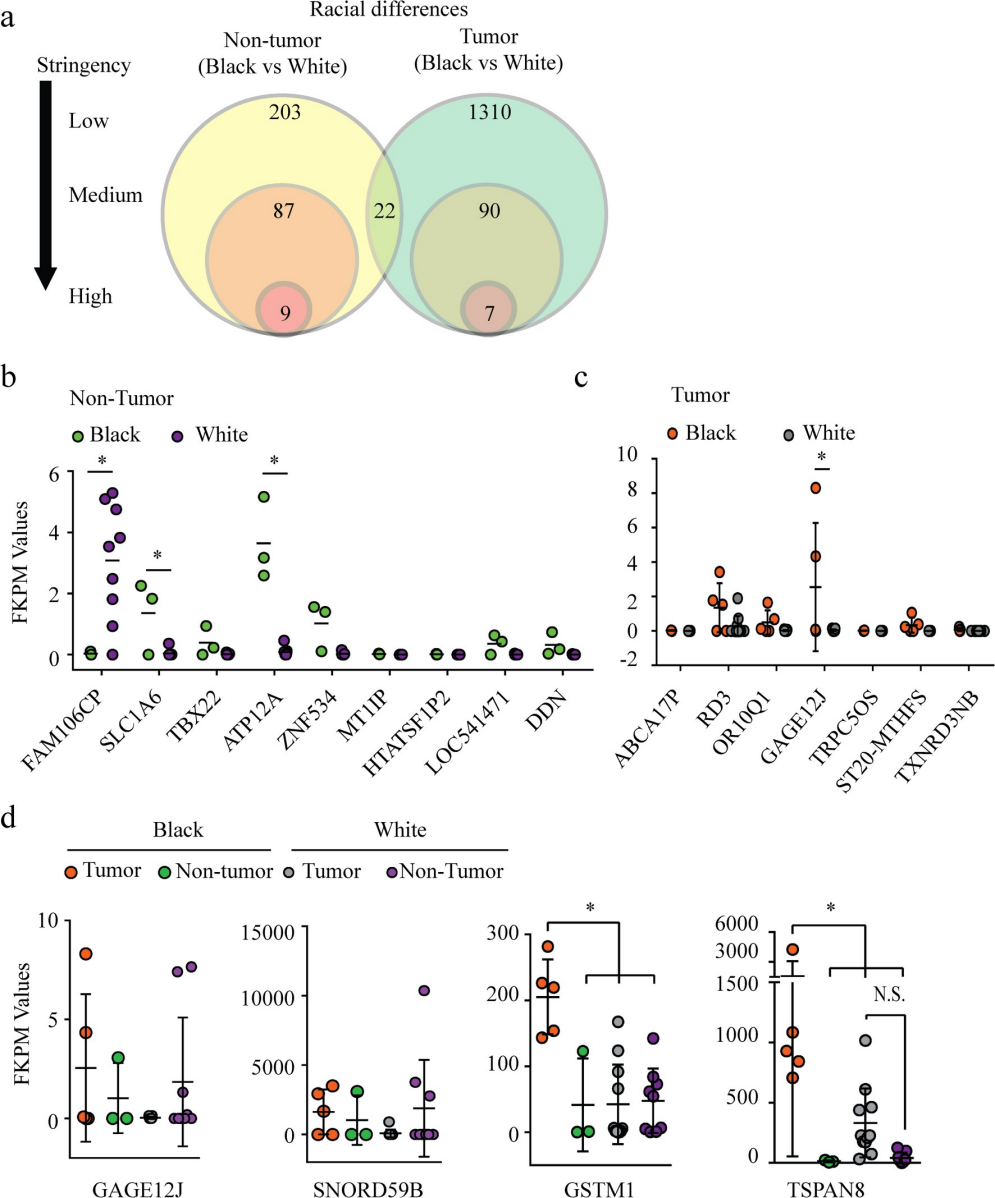
Characterization of genes expression differences between non-tumor and tumor pancreatic tissue from Black and White patients. (a) Venn diagram of the number of significant gene differences that meet the low, medium, and high stringency criteria using racially categorized non-tumor and tumor tissue. (b & c) Individual gene expression levels that met the high stringency criterion from different tissue types and racial backgrounds using non-tumor (b) and tumor tissue (c). A two-tailed ANOVA with tissue type and gene as factors with a Tukey’s post hoc test was leveraged to identify gene and tissue type interactions. (d) *GAGE12J*, *SNORD59B*, *GSTM1*, and *TSPAN8* expression levels in human pancreatic tumor and non-tumor tissue from Black and White patients. One way ANOVA was performed for each gene. * indicates a *p*<0.05.

To identify genetic differences in the pancreatic non-tumor tissue specimens associated with patient race, the gene signatures of pancreatic non-tumor tissues from Black and White patients were compared; using a Student’s T test with a 1% FDR, statistically significant differences in gene expression in non-tumor pancreatic tissue specimens obtained from Black patients (n = 3) and White patients (n = 9) were identified for 238 genes (S2 File). Among these genes, 92 met the medium stringency criterion (S2 Fig), and 9 met the high stringency criterion (Fig 3A). Using the 92 gene expression within the medium stringency criterion, individual gene expression levels in non-tumor pancreatic tissue from Black and White patients were compared using a two-way ANOVA test (Fig 3B and S2 Fig). Among the 9 genes that met the high stringency criterion, 3 genes were identified to have increased or decreased expression levels that appeared to be associated with race: *FAM106CP*, *SLC1A6*, and *ATP12A* (Fig 3B); however, the overall differences in expression levels of these genes were modest. Using a two-way ANOVA test of expression level differences between non-tumor tissue in Black patients and White patients for the 92 genes meeting the medium stringency criterion, *AMY1A* and *AMY2B* were statistically significantly elevated (S2 Fig).

To identify race-associated gene expression differences specific to the tumor tissue, pancreatic tumor tissue from Black Americans (n = 5) and White Americans (n = 11) were compared using a Student T test with a 1% FDR (S3 File). Among the 1310 genes differentially expressed in tumor tissue from Black and White patients, only 90 genes met the medium stringency criterion and 7 met the high stringency criterion (Fig 3A). Within this data, 22 genes were also shown to have race-associated differential expression in non-tumor pancreatic tissue. Using the individual genetic signatures of the pancreatic tissue specimens between races that met the medium and high stringency criterion, a two-way ANOVA test using race and gene as factors identified *GAGE12J*, *SNORD59B*, and *TSPAN8* as having increased expression in tumors from Black patients as compared to tumors from White patients (Fig 3C and S3 Fig). An increasing trend in *GSTM1* expression in tumors from Black patients as compared to tumors from White patients was also observed, although this trend was not statistically significant (S3 Fig).

To evaluate whether the differences in expression were associated with tissue type, a one-way ANOVA test was performed to assess whether expression of *GAGE12J*, *SNORD59B*, *GSTM1*, and *TSPAN8* was increased in pancreatic tumor tissue specimens from Black patients compared to tumor specimens from White patients and non-tumor specimens from both Black and White patients. According to this analysis, there was a statistically significant increase in level of expression of *GSTM1* and *TSPAN8*, but not *GAGE12J* and *SNORD59B* in the tumor tissue samples obtained from Black patients. Collectively, these preliminary data suggest that *GSTM1* and *TSPAN8* are upregulated in pancreatic tumors in Black patients we tested in comparison to non-tumorous pancreatic tissue in Black patients and either pancreatic tissue in White patients.

### Canonical pathway differences in pancreatic tumor tissue from Black and White patients

**Fig 4:**
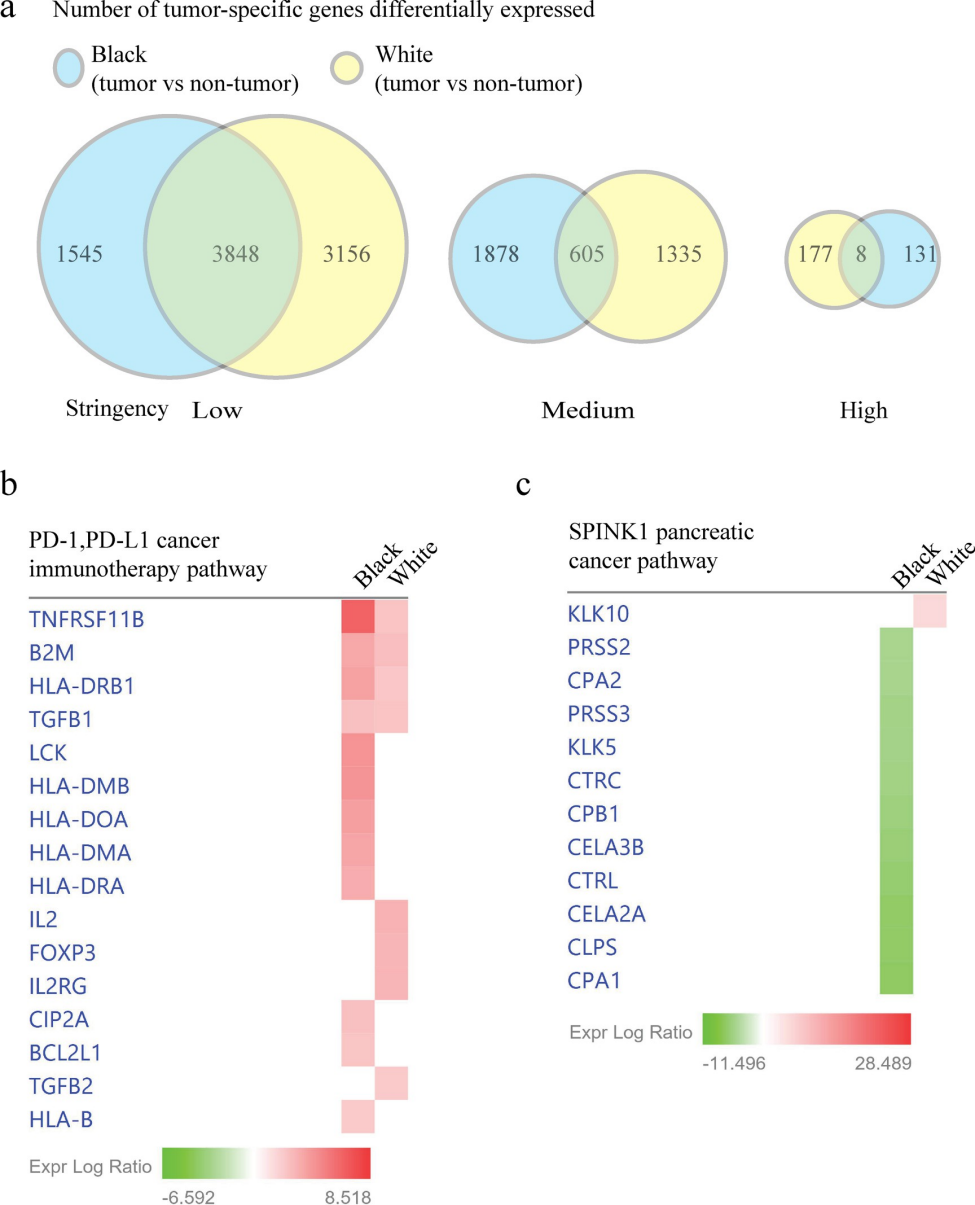
Pancreatic tissue gene expression differences with canonical pathway analyses between Black and White patients. (a) Number of tumor-specific genes with expression differences in pancreatic tumor as compared to non-tumor tissue from Black and White patients. (b) Pancreatic tumor and non-tumor tissue gene expression differences in the PD1, PD-L1 cancer immunotherapy pathway using genes that meet the medium stringency criterion from Black and White patients. (c) Gene expression differences in the SPINK1 pancreatic cancer pathway using genes that meet the high stringency criterion between pancreatic tumor and non-tumor tissue from Black and White patients.

Gene expression variation as a factor that may contribute to the pancreatic cancer racial incidence, severity, and outcomes disparities between Black and White patients, remains to be clearly established [5, 7] and requires further study. To evaluate the gene expression variation in pancreatic tumors from Black and White patients, the pancreatic tumor-specific gene expression profiles were compared to the pancreatic non-tumor tissue profiles for White patients (S4 File) and Black patients (S5 File). Using the genes identified to be significantly different by a Student’s T test with a 1% FDR (low stringency criterion), the number of genes with a significant difference in expression were quantified to be 1,545 and 3,156 from pancreatic tumors in Black and White patients, respectively (Fig 4A). Unexpectedly, the number of the genes differentially expressed in tumor tissue in Black patients increased to 1,878 genes under the medium stringency criterion as compared to the low stringency criterion, while the number of the tumor-associated genes samples from White patients decreased to 1,335 genes. This indicates the quantity of race-associative tumor-specific gene expression at a 2 < Log_2_ < -2-fold change was greater in Black patients as compared to White patients in this dataset. Using the high stringency criterion, only 8 genes were identified to have comparable differences in expression between Black and White patient non-tumor pancreatic tissue, while Black and White patient tumor-specific differences were observed for 177 and 131 genes, respectively.

The race-associated, tumor-specific gene expression differences identified from Black and White patients’ pancreatic non-tumor and tumor tissue were compared to reveal potential differences in canonical pathway regulation using IPA software [12]. Over 40 canonical pathways were identified to be statistically elevated or diminished in pancreatic tumor tissue from Black patients as compared to tissues from White patients, using the genetic differences identified under the medium stringency criterion (S4 Fig). In particular, the PD-1/ PD-L1 cancer immunotherapy pathway demonstrated an elevated expression ratio in 11 genes (*TNFRSF11B*, *B2M*, *HLA-DRB1*, *LCK*, *HLA-DMB*, *HLA-DOA*, *HLA-DMA*, *HLA-DRA*, *CIP2A*, *BCL2L1*, and *HLA-B*), and diminished expression ratios in 4 genes (*IL2*, *FOXP3*, *IL2RG*, and *TGFB2*) between tumor tissue from Black and White patients (Fig 4B). Using the genes that met the high stringency criterion, the only pathway identified to be differentially decreased and associated with race was the SPINK1 pancreatic cancer pathway, which had 11 genes (*PRSS2*, *CPA2*, *PRSS3*, *KLK5*, *CTRC*, *CPB1*, *CELA3B*, *CTRL*, *CELA2A*, *CLPS* and*CPA1)* downregulated in pancreatic tumor from Black patients as compared to tumor tissue from White patients (Fig 4C). SPINK1 is a pancreatic acinar cell specific gene and encodes a serine peptidase inhibitor (Kazal type 1) (Genbank: ID 6690).

### Gene expression and patient survival association

**Fig 5:**
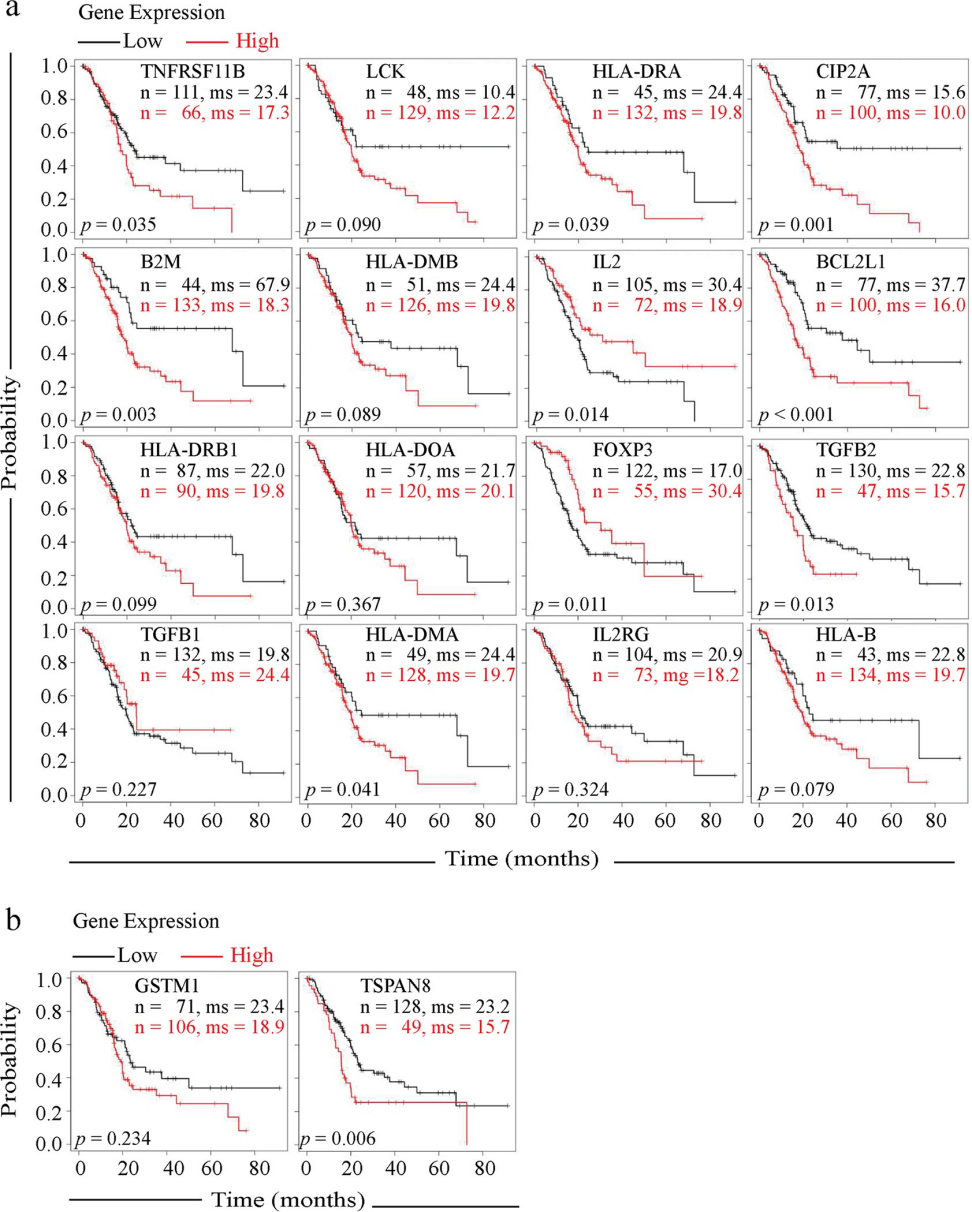
Gene expression level association with pancreatic cancer patient overall survival for 10 years. Kaplan-Meier plots of PD1 pathway gene expression difference between Black and White patients (a) and the race-associated and tumor specific GSTM1 and TSPAN8 genes from Black patients (b). The overall sample size (n) was 177. The median survival (ms) time in months and the log-ranked *p* value are provided for each analysis.

To evaluate an association between patient outcomes and gene expression differences, the differentially expressed genes between pancreatic tumors from Black and White patients in our dataset were used to assess whether there were statistically significant differences in the hazard ratio and median survival under low vs high gene expression levels using the overall 10-year patient survival and a pancreatic tumor patient database [13, 22]. Three (*B2M*, *CIP2A*, and *BCL2L1)* of the twelve genes that were identified as upregulated in pancreatic cancer tissue from Black patients as compared to White patients that are associated with the PD1 pathway (Fig 4B) were associated with an increase in the hazard ratio (ratio > 2) for overall survival and a decrease in median survival (Fig 5A and S6 Table). Higher expression of these genes above the software-established threshold was associated with a decrease in median survival time by 49.6 months for *B2M*, 5.6 months for *CIP2A*, and 21.7 months for *BCL2L1*. Among the four downregulated genes in the PD1 pathway of Black patients as compared to White patients (Fig 4B), *IL2* and *FOXP3* expression below the set threshold revealed a decrease in median overall survival by 11.5 and 13.4 months, respectively. In contrast to *GSTM1*, *TSPAN8* was the only upregulated Black patient tumor specific gene (Fig 3C) that at high expression levels associated with an increase in the hazard ratio (1.81) and decrease overall survival by 7.5 months (Fig 5B).

## Discussion

This report provides exploratory transcriptomic insights into pancreatic tumor and non-tumor tissue from Black and White patients. These data provide insight into differences in gene expression associated with race which illustrates the need for diverse cancer genetic databases in order to elucidate potential genomic factors contributing to cancer health disparities. Using this validated dataset, we identified potential gene targets that may be used for the development of therapeutics and biomarker assays. In addition, we characterized differentially expressed genes in non-tumor and tumor pancreatic tissues to provide early supportive evidence of differential gene expression in the pancreatic tissue of Black patients as compared to White patients. Differences in molecular profiles of tumors from diverse populations may explain differences in incidence and severity of the disease, and/or may predict for variable response to anti-cancer treatments.

A limitation of our study is its relatively small sample size and as such, further studies will be needed to confirm our findings. One aim of our report is that these findings generate new evidence-based hypotheses for follow-up clinical studies with a large and diverse patient population focused on potential contributors to differences in outcomes of patients with pancreatic cancer, pre-clinical studies that are investigating potential proteins with therapeutic potential for pancreatic cancer, and assay developments that are identifying new potential diagnostic biomarkers.

To identify target genes and proteins as biomarkers that may improve pancreatic cancer outcomes, over 4,000 genes differentially expressed in tumor and non-tumor tissue were categorized using a selection criterion based on significant (*p* <0.05) differences and expression level changes (S2 Table). Using this approach and verifying the data using individual gene expression data, we found that *AGR2*, *CEACAM6*, *GNMT*, *PDIA2*, *POSTN*, *RBPJL*, and *S100P* are upregulated in pancreatic tumor tissue as compared to non-tumor tissue from Black and White patients (Fig 1). The protein products from *AGR2* [16, 23–25], *CEAMAN6* [26–28], *GNMT* [29, 30], *PDIA2* [31, 32], *POSTN* [17, 33, 34], *RBPJL* [35, 36] and *S100P* [37–39] have been reported as potential diagnostic and prognostic biomarkers for pancreatic cancer or have demonstrated to be involved in either pancreatic cancer, initiation, migration, invasion, metastasis, or chemoresistance. This report provides additional evidence to support that these genes are specifically upregulated in pancreatic tumor tissue as compared to non-tumor tissue and their products could potentially serve as differentiating biomarkers in pancreatic cancer.

To our knowledge, no prior studies have focused on PD1/PDL-1 immunotherapy pathway differences between races or the race-specific outcomes for patients that receive immune checkpoint inhibitors for pancreatic cancer. In 2017, the Food and Drug Administration granted accelerated approval to the immune checkpoint inhibitor pembrolizumab for the treatment of mismatch repair-deficient and microsatellite instability-high (dMMR/MSI-H) refractory solid tumors based on efficacy results showing an objective response rate of 36.9%, including a complete response rate of 7% [40–42] in 149 patients with a variety of dMMR/MSI-H solid tumors, including 6 patients with pancreatic cancer. In a subsequent study, reported objective response rates were lower (18.2%) among 22 patients with pancreatic cancer enrolled in the trial [40, 43], and effective immunotherapy to treat most patients with pancreatic cancer remains an unmet need. Although reported response rates to pembrolizumab in the overall population with MSI-H/dMMR pancreatic cancer vary; it is not known whether response rates to immune checkpoint inhibition differs in Black and White patients. Our exploratory findings suggest that the immune checkpoint inhibition pathway may be upregulated in Black patients, which could theoretically cause differences in treatment responses. Additional pharmacogenomic studies with a larger population of Black and White participants with additional patient baseline demographic and pancreatic cancer-specific information, such as history of germ-line cancer syndromes and location of biopsies, are needed to further assess and confirm the differences observed in our dataset. Further studies are warranted to explore potential differences in PD-L1 status of these specimens and whether differential upregulation of the PD-1/PD-L1 pathway impacts outcomes in Black and White patients.

Non-tumor pancreatic tissue from Black and White patients were characterized to reveal modest differences in expression of 238 genes, while the pancreatic tumor tissue had 1310 genes that were expressed differently between Black and White patients under the low stringency criterion (Fig 3). Using the high stringency criterion and individual gene expression profiles, *GSTM1* and *TSPAN8* were identified to be potential tumor-specific genes that were uniquely upregulated in pancreatic tumor tissue from Black patients as compared to the non-tumor tissue from Black patients and both tumor and non-tumor tissue from White patients. The gene product of TSPAN8 has been implicated in regulating metastasis in pancreatic cancer and is a proposed target candidate for immunotherapy for pancreatic cancer [44, 45]. However, the role of the *GSTM1* gene product is only associated with a risk of developing pancreatic cancer and the mechanistic role remains unclear [46], and warrants further investigation with targeted mechanistic studies on GSTM1 protein. Collectively, our data suggest that the presence of key genetic differences may be one potential contributing factor to the observed disparities in pancreatic cancer mortality between Black and White patients.

We recognize that race is an imperfect proxy for biological differences, including gene expression, across the population. A limitation of our study is that we did not examine associated gene expression in the context of genetic ancestry. Additionally, many other factors could impact gene expression such as age, past medical history including prior chemotherapy and radiation, germ-line cancer syndromes, biopsy location, and others. We also acknowledge additional caveats with respect to our findings, including lack of control for multiplicity, and that other intrinsic and extrinsic factors such as comorbidities, tumor PD-L1 status and presence of other oncogenic drivers, and socioeconomic factors, can contribute to differences in overall survival in patients with pancreatic cancer. Therefore, larger studies which evaluate multiple factors are needed to confirm our findings and provide a stronger correlation with cancer outcomes.

## Conclusions

Patients with pancreatic cancer have a high unmet medical need for effective treatments. New treatments should be evaluated in the context of a better understanding of the contributing factors leading to racial disparities in disease incidence and severity with the aim of improving outcomes for all patients. Here, we provide a pancreatic cancer dataset and analyses that compared the transcriptomics of tumor and non-tumor pancreatic tissues from Black and White patients. Using this dataset, we identified *AGR2*, *CEAMAN6*, *GNMT*, *PDIA2*, *POSTN*, *RBPJL*, and *S100P* genes to be associated with pancreatic tumor tissue as compared to non-tumor tissue that were irrespective of race, and *TSPAN8* and *GSTM1* as potential pancreatic tumor-specific upregulated genes in pancreatic cancer tissue from Black patients as compared to non-tumor pancreatic tissue in Black patients and both pancreatic tumor and non-tumor tissues from White patients. We propose that collectively, these datasets and exploratory findings can be leveraged to conduct more comprehensive studies to enable a better understanding of differences in pancreatic tumor biology among different races to facilitate personalized medical approaches to diagnosis, treatment and ultimately improve patient outcomes.

## Supporting information

S1 Fig FKPM values of genes from human pancreatic tumor and non-tumor tissue. Genes identified to be statistically different (p<0.05 with 1% false discovery rate) between tissue specimens with an expression fold change of 5 < Log2 < -5.
(TIF)

S2 Fig FKPM values of genes from Black and White human pancreatic non-tumor tissue. Genes identified to be statistically different (p<0.05 with 1% false discovery rate) between tissue specimens with an expression fold change of 2 < Log2 < -2.
(TIF)

S3 Fig FKPM values of genes from Black and White human pancreatic non-tumor tissue. Genes identified to be statistically different (p<0.05 with 1% false discovery rate) between tissue specimens with an expression fold change of 2 < Log2 < -2.
(TIF)

S4 Fig IPA of genetic differences. Using IPA software, the tumor specific differences in the canonical pathways from Black and White pancreatic tissue was compared to identify pathways with a z score greater than 1.7 using the genes identified under medium stringency criterion. White blocks indicate the genetic pathway differences did not meet medium stringency criterion.
(TIF)

S1 Table Type of analysis, degree of freedom and figure location for each statistical analysis within the manuscript. (DOCX)

S2 Table Description of subject, tissue harvested and tumor stage. (DOCX)

S3 Table Number of genes identified in each stringency criteria. (DOCX)

S4 Table Protein location discrepancies between the Uniprot and IPA databases. (DOCX)

S5 Table Localization of theoretical proteins from genes identified to be significantly different in pancreatic tumor tissue as compared to non-tumor pancreatic tissue. (DOCX)

S6 Table Hazard ratio with range and expression range with threshold setting for patient survival using the Kalpan-Meier curve analysis. (DOCX)

S1 File Gene comparison between non-tumor and tumor tissue from patients. (XLSX)

S2 File Gene comparison between non-tumor tissue from Black and White patients. (XLSX)

S3 File Gene comparison between tumor tissue from Black and White patients. (XLSX)

S4 File Gene comparison between non-tumor and tumor tissues from White patients. (XLSX)

S5 File Gene comparison between non-tumor and tumor tissues from Black patients. (XLSX)
