# Supplementary figures and images for: Transcriptomic analysis of pancreatic adenocarcinoma specimens obtained from Black and White patients
Source: PLoS One. 2023 Feb 22;18(2):e0281182. doi: 10.1371/journal.pone.0281182 (PMC9946261; doi:10.1371/journal.pone.0281182)

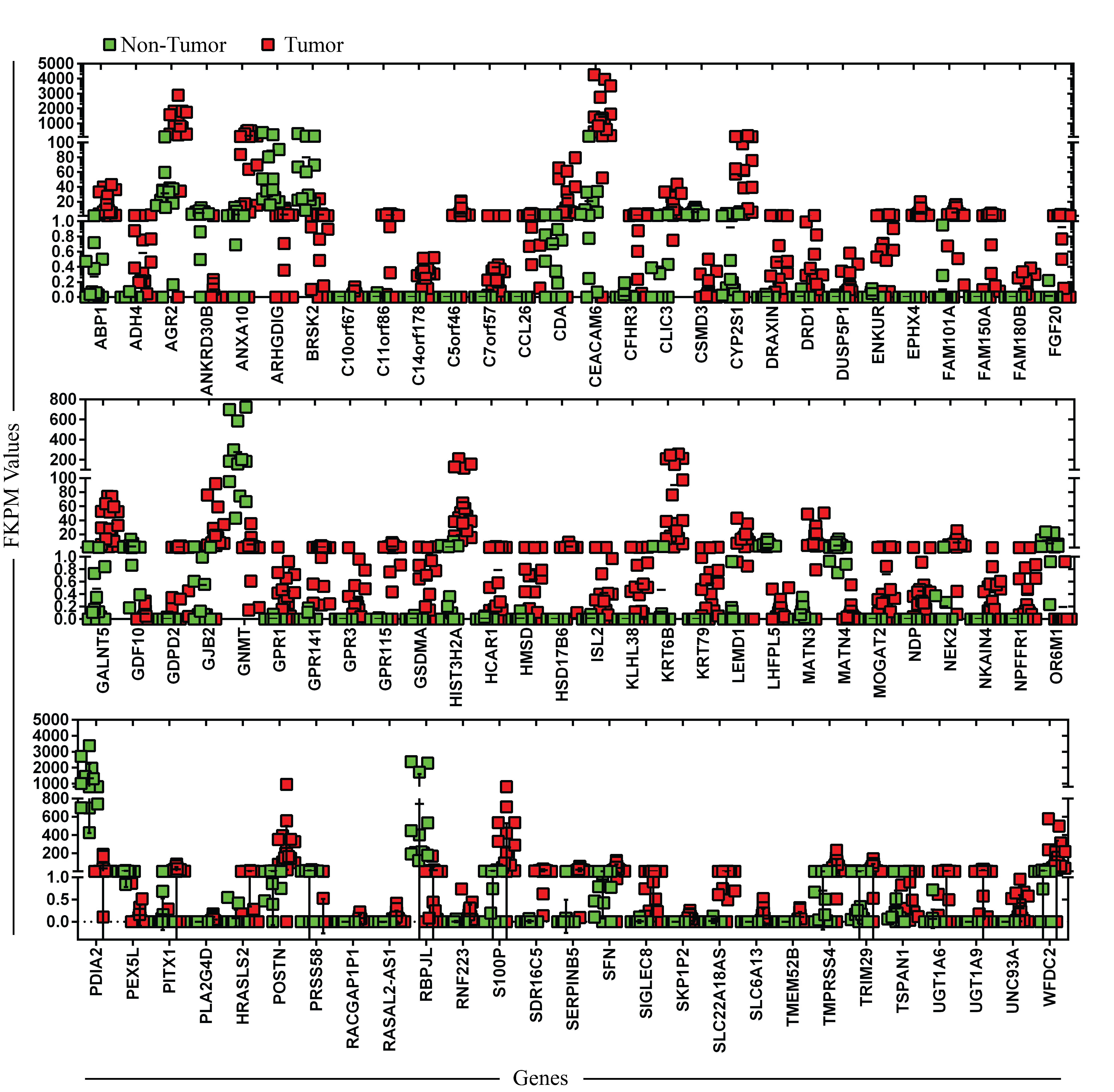

Supplement: S1 Fig — Genes identified to be statistically different (p<0.05 with 1% false discovery rate) between tissue specimens with an expression fold change of 5 < Log2 < -5. (TIF) [file pone.0281182.s001.tif]

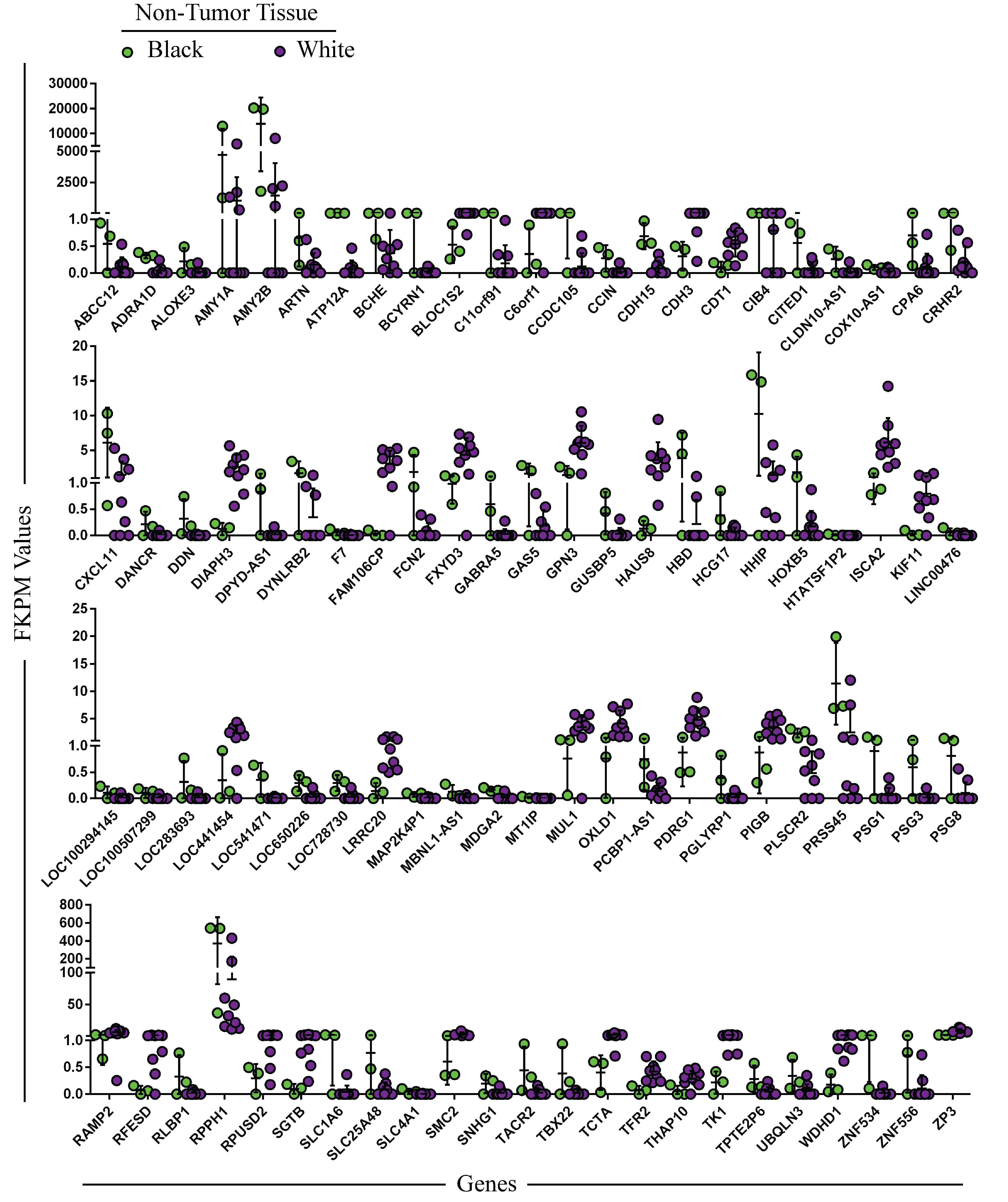

Supplement: S2 Fig — Genes identified to be statistically different (p<0.05 with 1% false discovery rate) between tissue specimens with an expression fold change of 2 < Log2 < -2. (TIF) [file pone.0281182.s002.tif]

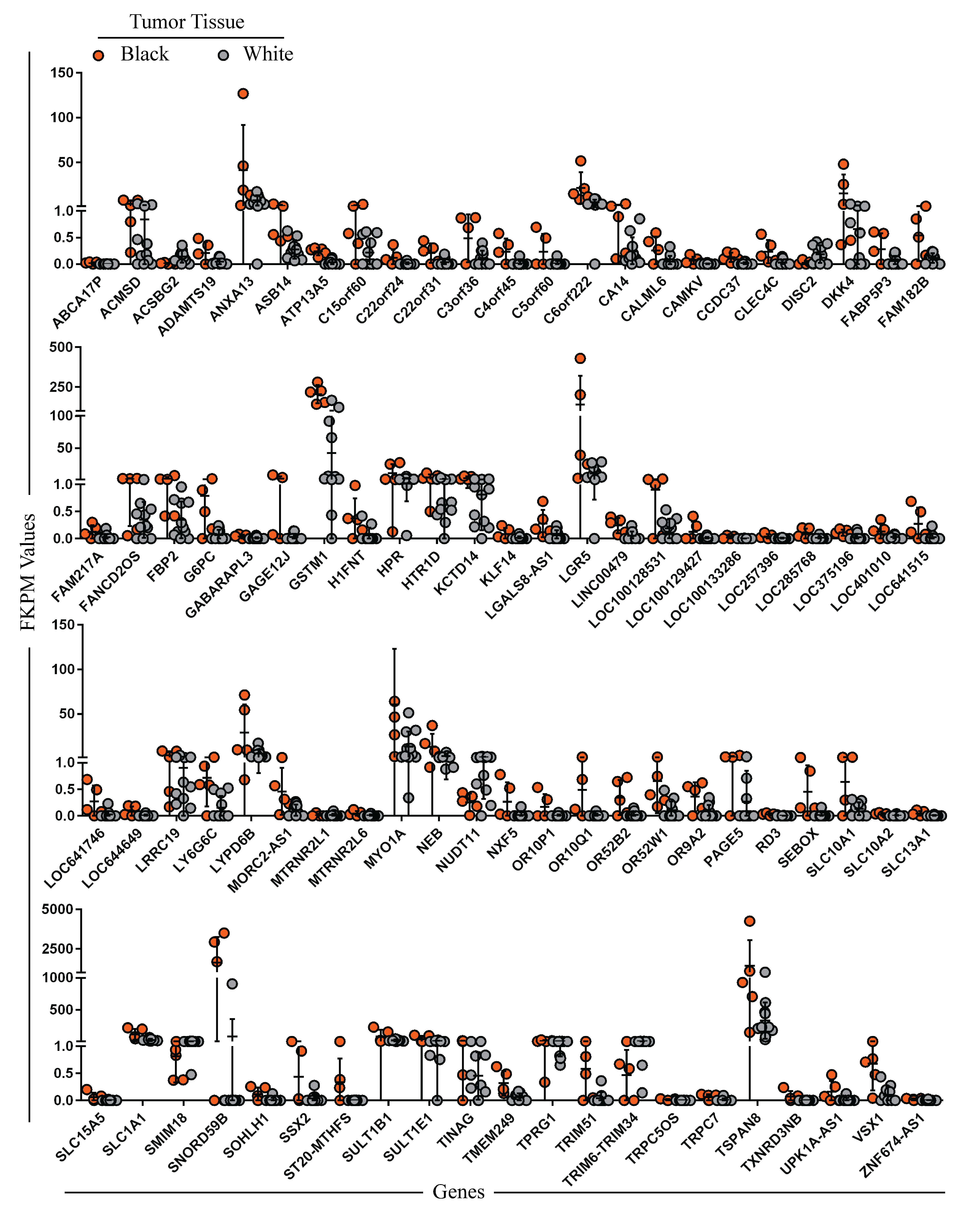

Supplement: S3 Fig — Genes identified to be statistically different (p<0.05 with 1% false discovery rate) between tissue specimens with an expression fold change of 2 < Log2 < -2. (TIF) [file pone.0281182.s003.tif]

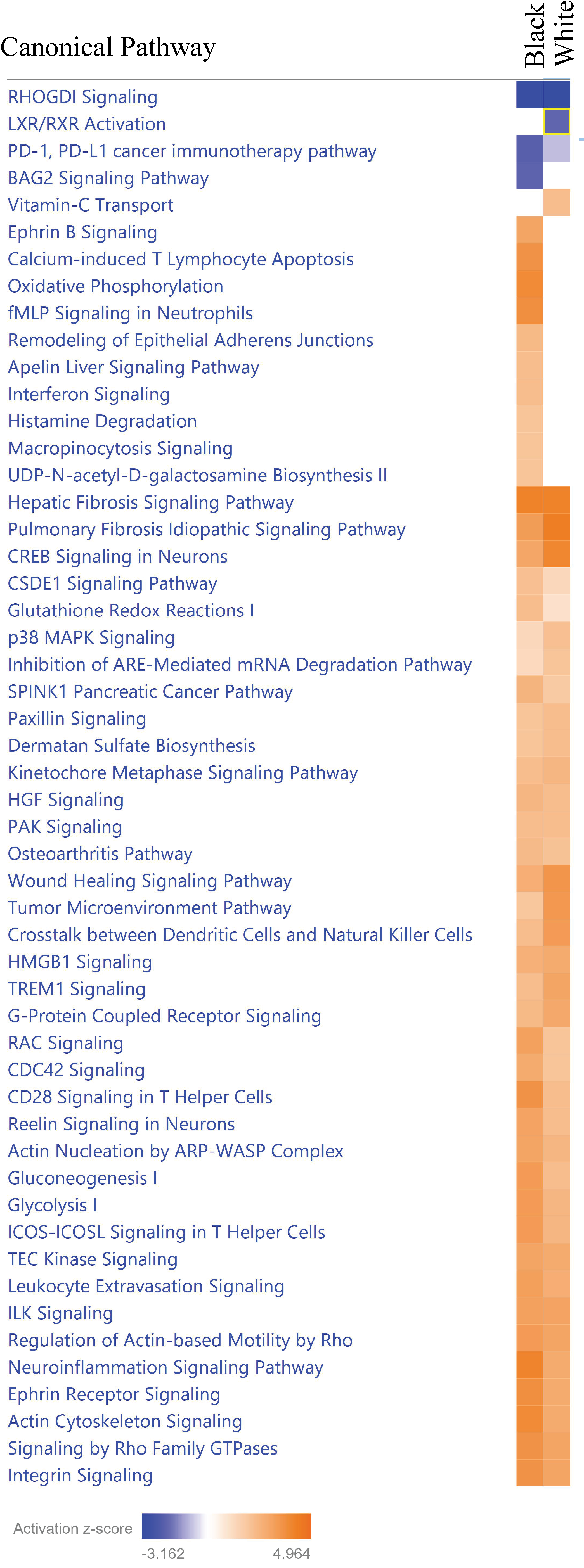

Supplement: S4 Fig — Using IPA software, the tumor specific differences in the canonical pathways from Black and White pancreatic tissue was compared to identify pathways with a z score greater than 1.7 using the genes identified under medium stringency criterion. White blocks indicate the genetic pathway differences did not meet medium stringency criterion. (TIF) [file pone.0281182.s004.tif]
