# Supplementary material for: Transcriptomic analysis of pancreatic adenocarcinoma specimens obtained from Black and White patients
Source: PLoS One. 2023 Feb 22;18(2):e0281182. doi: 10.1371/journal.pone.0281182 (PMC9946261; doi:10.1371/journal.pone.0281182)
Supplement: S1 Table — (DOCX) [file pone.0281182.s005.docx]

| **Two Way ANOVA** | | | | | | | |
| --- | --- | --- | --- | --- | --- | --- | --- |
| Figure | | Interaction and Factors | | DF | MS | F (DFn, DFd) | *p* value |
| Figure 1B and Supplemental Figure 1 | | Interaction | | 83 | 453641 | F (83, 2184) = 15.94 | < 0.0001 |
|  |  | Gene | | 83 | 450527 | F (83, 2184) = 15.83 | < 0.0001 |
|  |  | Tissue Type | | 1 | 135800 | F (1, 2184) = 4.773 | 0.0290 |
| Supplementary Figure 2 | | Interaction | | 91 | 4E+06 | F (91, 920) = 9.305 | < 0.0001 |
|  |  | Gene | | 91 | 7E+06 | F (91, 920) = 14.68 | < 0.0001 |
|  |  | Race | | 1 | 7E+06 | F (1, 920) = 14.97 | 0.0001 |
| Figure 3b | | Interaction | | 8 | 6.572 | F (8, 90) = 15.72 | < 0.0001 |
|  |  | Gene | | 8 | 4.221 | F (8, 90) = 10.10 | < 0.0001 |
|  |  | Race | | 1 | 3.818 | F (1, 90) = 9.135 | 0.0033 |
| Figure 4b | | Interaction | | 6 | 2.811 | F (6, 97) = 3.893 | 0.0016 |
|  |  | Gene | | 6 | 3.349 | F (6, 97) = 4.638 | 0.0003 |
|  |  | Tissue Type | | 1 | 9.932 | F (1, 97) = 13.75 | 0.0003 |
| Supplementary Figure 3 | | Interaction | | 89 | 135677 | F (89, 1259) = 7.448 | < 0.0001 |
|  |  | Gene | | 89 | 227736 | F (89, 1259) = 12.50 | < 0.0001 |
|  |  | Race | | 1 | 422474 | F (1, 1259) = 23.19 | < 0.0001 |
|  | | | | | | | |
| **One Way ANOVA** | | | | | | | |
| Figure 4c -Tissue Type |  | | Gene | DF | MS | F (DFn, DFd) | *p* value |
|  | Tissue Type | | GAGE12J | 3 | 9.264 | F (3, 24) = 1.515 | 0.2361 |
|  |  |  | SNORD59B | 3 | 6E+06 | F (3, 24) = 1.257 | 0.3113 |
|  |  |  | GSMT1 | 3 | 35419 | F (3, 24) = 10.85 | 0.0001 |
|  |  |  | TSPAN8 | 3 | 3E+06 | F (3, 24) = 6.757 | 0.0018 |
