# Supplementary material for: Transcriptomic analysis of pancreatic adenocarcinoma specimens obtained from Black and White patients
Source: PLoS One. 2023 Feb 22;18(2):e0281182. doi: 10.1371/journal.pone.0281182 (PMC9946261; doi:10.1371/journal.pone.0281182)
Supplement: S2 Table — (DOCX) [file pone.0281182.s006.docx]

| ***Tissue type, origin, and quantity*** | | | |
| --- | --- | --- | --- |
| *Tissue* | *Ethnicity* | *Disease characteristics* | *Number and percent of  specimen per race* |
| Pancreatic ductal  adenocarcinoma (Tumor) | White | T1N0 | 1 (9%) |
|  | White | T2N1 | 1 (9%) |
|  | White | T2N2 | 1 (9%) |
|  | White | *y*T3N1 | 1 (9%) |
|  | White | T3N1 | 7 (64%) |
|  | Black | T2N2 | 1 (20%) |
|  | Black | T2N2 | 1 (20%) |
|  | Black | *y*T3N0 | 1 (20% |
|  | Black | T2N2 | 1 (20%) |
|  | Black | T3N1 | 1 (20%) |
| Non-tumor | White | Pancreatitis | 5 (56%) |
|  | White | Tumor free tissue | 2 (22%) |
|  | White | Mucinous cystic neoplasm | 1 (11%) |
|  | White | Mucinous cystadenoma | 1 (11%) |
|  | Black | Chronic pancreatitis | 1 (33%) |
|  | Black | Pancreatitis | 2 (67%) |
