# Supplementary material for: Transcriptomic analysis of pancreatic adenocarcinoma specimens obtained from Black and White patients
Source: PLoS One. 2023 Feb 22;18(2):e0281182. doi: 10.1371/journal.pone.0281182 (PMC9946261; doi:10.1371/journal.pone.0281182)
Supplement: S3 Table — (DOCX) [file pone.0281182.s007.docx]

| Transcriptomic Dataset Stringency Criteria, Pre-defined Statistical Thresholds, and Results. | | | | | | | | |
| --- | --- | --- | --- | --- | --- | --- | --- | --- |
| Stringency | *p* value | *1% False Discovery Rate* | *Log2 fold change Restrictions* | Genes identified | | | | |
|  |  |  |  | Tumor vs Non-Tumor | Non-Tumor (Black vs White) | Tumor  (Black vs White) | Black  (Tumor vs Non-tumor) | White (Tumor vs Non-tumor) |
| Low | <0.05 | Yes | None | 4665 | 238 | 1357 | 7212 | 5655 |
| Medium | <0.05 | Yes | 2 < Log2 < -2 | 1300 | 92 | 90 | 1977 | 2524 |
| High | <0.05 | Yes | 5 < Log2 < -5 | 84 | 9 | 7 | 144 | 188 |
