# Supplementary material for: Transcriptomic analysis of pancreatic adenocarcinoma specimens obtained from Black and White patients
Source: PLoS One. 2023 Feb 22;18(2):e0281182. doi: 10.1371/journal.pone.0281182 (PMC9946261; doi:10.1371/journal.pone.0281182)
Supplement: S4 Table — (DOCX) [file pone.0281182.s008.docx]

|  |  | Uniprot Database Results | | | | |  | IPA Results |
| --- | --- | --- | --- | --- | --- | --- | --- | --- |
| Gene ID | Expr Log Ratio | ID | Intracellular | Plasma membrane | Secreted | Not Provided |  | Location |
| ANKRD30B | -5.535 | Q9BXX2 |  |  |  | x |  | Extracellular Space |
| PM20D1 | -5.26 | Q6GTS8 |  |  | x |  |  | Cytoplasm |
| RNF223 | 5.105 | E7ERA6 | x |  |  |  |  | Other |
| FAM101A | 5.46 | Q6ZTI6 | x |  |  |  |  | Other |
| HMSD | 5.733 | A8MTL9 |  |  | x |  |  | Other |
| DUSP5P1 | 5.945 | Q12874 | x | x |  |  |  | Other |
| SLC22A18AS | 6.561 | Q8N1D0 |  |  |  | x |  | Other |
| TMPRSS4 | 6.601 | Q9NRS4 |  | x |  |  |  | Cytoplasm |
| C11orf86 | 8.078 | A6NJI1 |  |  |  | x |  | Other |
| C10orf67 | 9.104 | Q8IYJ2 | x |  |  |  |  | Other |
| FAM180B | 10.306 | Q6P0A1 |  |  | x |  |  | Other |
| KRT79 | 12.491 | Q5XKE5 | x |  |  |  |  | Extracellular Space |
| FAM150A | 13.892 | Q6UXT8 |  |  | x |  |  | Other |
| EPHX4 | 15.967 | Q8IUS5 | x |  |  |  |  | Other |
| RASAL2-AS1 | 8.234 |  |  |  |  |  |  | Other |
| RACGAP1P | 9.682 |  |  |  |  |  |  | Other |
| SKP1P2 | 10.073 | Not Provided |  |  |  | x |  | Other |
| C14orf178 | 11.145 | Q8N769 |  |  |  | x |  | Other |
| C7orf57 | 11.943 | Q8NEG2 |  |  |  |  |  | Other |
| KLHL38 | 12.474 | Q2WGJ6 |  |  |  | x |  | Other |
| PRSS58 | -5.487 | Q8IYP2 |  |  | x |  |  | Other |
| NKAIN4 | 11.869 | Q8IVV8 |  | x |  |  |  | Other |
| HSD17B6 | 14.737 | O14756 | x |  |  |  |  | Other |
| C5orf46 | 15.913 | Q6UWT4 |  |  | x |  |  | Cytoplasm |
