# Supplementary material for: Transcriptomic analysis of pancreatic adenocarcinoma specimens obtained from Black and White patients
Source: PLoS One. 2023 Feb 22;18(2):e0281182. doi: 10.1371/journal.pone.0281182 (PMC9946261; doi:10.1371/journal.pone.0281182)
Supplement: S5 Table — (DOCX) [file pone.0281182.s009.docx]

| Gene ID | Protein Name | Expr Log Ratio | ID | Organelle | Plasma membrane | Secreted |
| --- | --- | --- | --- | --- | --- | --- |
| CSMD3 | CUB and sushi domain-containing protein 3 | -5.9 | Q7Z407 |  | x |  |
| RBPJL | Recombining binding protein suppressor of hairless-like protein | -5.8 | Q9UBG7 | Nucleus |  |  |
| PDIA2 | Protein disulfide-isomerase A2 | -5.7 | Q13087 | Endoplasmic Reticulum |  |  |
| CLIC3 | Chloride intracellular channel protein 3 | -5.3 | O95833 | Nucleus |  |  |
| AGR2 | Anterior gradient protein 2 homolog | -5.0 | O95994 | Endoplasmic Reticulum |  | x |
| HIST3H2A | Histone H2A type 3 | -5.0 | Q7L7L0 | Nucleus |  |  |
| TRIM29 | Tripartite motif-containing protein 29 | -5.0 | Q14134 | Lysosome |  |  |
| CDA | Cytidine deaminase | 5.0 | P32320 |  |  | x |
| NEK2 | Never in mitosis A-related kinase 2 | 5.0 | P51955 | Nucleus |  |  |
| TSPAN1 | Tetraspanin-1 | 5.0 | O60635 | Lysosome |  |  |
| DRD1 | D(1A) dopamine receptor | 5.0 | P21728 | Endoplasmic reticulum | x |  |
| SFN | 14-3-3 protein sigma | 5.1 | P31947 | Nucleus |  | x |
| ISL2 | Insulin gene enhancer protein ISL | 5.2 | Q96A47 | Nucleus |  |  |
| UGT1A6 | UDP-glucuronosyltransferase 1-6 | 5.2 | P19224 | Endoplasmic Reticulum |  |  |
| CYP2S1 | Cytochrome P450 2S1 | 5.3 | Q96SQ9 | Endoplasmic Reticulum |  |  |
| GALNT5 | Polypeptide N-acetylgalactosaminyltransferase 5 | 5.3 | Q7Z7M9 | Golgi Apparatus |  |  |
| POSTN | Periostin | 5.3 | Q15063 | Golgi Apparatus |  | x |
| S100P | Protein S100-P | 5.4 | P25815 | Nucleus |  |  |
| PITX1 | Pituitary homeobox 1 | 5.4 | P78337 | Nucleus |  |  |
| UGT1A10 | UDP-glucuronosyltransferase 1A10, UGT1A10 | 5.8 | Q9HAW8 | Endoplasmic Reticulum |  |  |
| GDPD2 | Glycerophosphoinositol inositolphosphodiesterase | 5.9 | Q9HCC8 |  | x |  |
| SDR16C5 | Epidermal retinol dehydrogenase 2 | 6.0 | Q8N3Y7 | Endoplasmic Reticulum |  |  |
| MOGAT2 | 2-acylglycerol O-acyltransferase 2 | 6.2 | Q3SYC2 | Endoplasmic Reticulum |  |  |
