# Supplementary material for: Transcriptomic analysis of pancreatic adenocarcinoma specimens obtained from Black and White patients
Source: PLoS One. 2023 Feb 22;18(2):e0281182. doi: 10.1371/journal.pone.0281182 (PMC9946261; doi:10.1371/journal.pone.0281182)
Supplement: S6 Table — (DOCX) [file pone.0281182.s010.docx]

| GENE ID | Hazard ratio (HR) | HR Range | Expression Range | Threshold |
| --- | --- | --- | --- | --- |
| TNFRSF11B | 1.56 | 1.03 to 2.35 | 1 to 5332 | 485 |
| B2M | 2.19 | 1.29 to 3.71 | 8661 to 200161 | 58245 |
| HLA-DRB1 | 1.42 | 0.94 to 2.15 | 334 to 52433 | 11130 |
| TGFB1 | 0.73 | 0.43 to 1.23 | 143 to 7763 | 2898 |
| LCK | 1.54 | 0.93 to 2.53 | 11 to 4573 | 219 |
| HLA-DMB | 1.5 | 0.94 to 2.39 | 53 to 6698 | 845 |
| HLA-DOA | 1.22 | 0.79 to 1.91 | 25 to 5401 | 541 |
| HLA-DMA | 1.65 | 1.02 to 2.69 | 115 to 8116 | 1611 |
| HLA-DRA | 1.68 | 1.02 to 2.75 | 606 to 95464 | 10020 |
| IL2 | 0.58 | 0.37 to 0.9 | 0 to 28 | 1 |
| FOXP3 | 0.53 | 0.32 to 0.87 | 4 to 1014 | 123 |
| IL2RG | 1.23 | 0.81 to 1.86 | 15 to 10542 | 2089 |
| CIP2A | 2.08 | 1.32 to 3.27 | 10 to 463 | 119 |
| BCL2L1 | 2.15 | 1.38 to 3.36 | 1121 to 13415 | 4055 |
| TGFB2 | 1.74 | 1.12 to 2.71 | 9 to 4389 | 648 |
| HLA-B | 1.58 | 0.94 to 2.64 | 4551 to 166868 | 33755 |
| GSTM1 | 1.29 | 0.85 to 1.98 | 0 to 2781 | 8 |
| TSPAN8 | 1.81 | 1.18 to 2.77 | 1 to 92704 | 12108 |
